# The effect of DASH diet on atherogenic indices, pro-oxidant-antioxidant balance, and liver steatosis in obese adults with non-alcoholic fatty liver disease: A double-blind controlled randomized clinical trial

**DOI:** 10.34172/hpp.2023.10

**Published:** 2023-04-30

**Authors:** Taghi Badali, Sara Arefhosseini, Farnaz Rooholahzadegan, Helda Tutunchi, Mehrangiz Ebrahimi-Mameghani

**Affiliations:** ^1^Student Research Committee, Tabriz University of Medical Sciences, Tabriz, Iran; ^2^Endocrine Research Center, Tabriz University of Medical Sciences, Tabriz, Iran; ^3^Nutrition Research Center, Department of Biochemistry and Diet Therapy, Faculty of Nutrition and Food Sciences, Tabriz University of Medical Sciences, Tabriz, Iran

**Keywords:** Clinical trial, Dietary approaches to stop hypertension, Non-alcoholic fatty liver disease, Obesity

## Abstract

**Background:** The present clinical trial aimed to examine whether adherence to Dietary Approaches to Stop Hypertension (DASH) diet could improve lipid profile, the Pro-oxidant-antioxidant balance (PAB) as well as liver function in obese adults with non-alcoholic fatty liver disease (NAFLD).

**Methods:** Sixty two patients with NAFLD were equally allocated into either DASH or low-calorie diet (LCD) group for 8 weeks. The primary and secondary outcomes were determined before and after the trial.

**Results:** Forty patients completed the trial. Significant within group differences were found in dietary saturated fat, selenium, vitamins A and E as well as body weight and body mass index (BMI) and waist circumference (WC) after the intervention (*P*<0.05). DASH diet showed greater significant change in systolic and diastolic blood pressure without significant differences between the groups after 8 weeks. Apart from serum high-density lipoprotein cholesterol (HDL-C) and triglyceride/HDL-C, greater reductions were found not only in serum lipids and atherogenic indices (*P*<0.05) but also in serum aspartate aminotransferase (AST), AST to platelet ratio index (APRI) and lipid accumulation product (LAP) in DASH group in comparison to control group (*P*=0.008, *P*=0.019 and *P*=0.003, respectively). Nevertheless, there was not any difference in PAB level between the groups. Furthermore, adherence to DASH diet was more effective in alleviating liver steatosis compared with usual LCD (*P*=0.012).

**Conclusion:** Adherence to DASH diet appears to be more effective in improving obesity, atherogenic and liver steatosis biomarkers but not oxidative stress (OS) than usual LCD.

## Introduction

 Non-alcoholic fatty liver disease (NAFLD) is considered as the most common liver disease affecting around one billion people worldwide, ranged from simple steatosis to fibrosis, cirrhosis and hepatocarcinoma.^1^ There is accumulating evidence supporting the “Multi-hit” model in the pathophysiology of NAFLD, particularly in a close relationship with the components of metabolic syndrome (MetS).^2^ Insulin resistance (IR) is strongly associated with NAFLD.^3^ IR causes multiple alterations leading to disturbances in mitochondrial betaoxidation and free radicals production in both gut and adipose tissue, and in turn, oxidative stress (OS).^4,5^ In hepatic cells, mitochondrial activities regulate energy and fat homeostasis.^4^ These activities include electron transfer, β-oxidation of free fatty acids (FFAs) and also the production of reactive oxygen species (ROS).^6^ Therefore, because of the impairment in pro-oxidant and antioxidant balance and also blocking fatty acid (FA) β-oxidation, FAs increase and ROS are produced, and in turn, causes OS.^7,8^

 OS – a major factor in NAFLD pathogenesis – has been assessed by ROS levels and lipid peroxidation products.^7^ Nevertheless, studies investigating antioxidant status among patients with NAFLD have reported conflicting results.^7,9,10^ On the other hand, most studies have separately determined the total oxidant and antioxidant status. Pro-oxidants/antioxidants balance (PAB) – assessed as pro-oxidants/antioxidants ratio in one test – appears to be better indicator of both oxidants and antioxidant condition.^11^

 Despite considerable clinical research, no approved treatments have been suggested for NAFLD.^12^ To date, therapy interventions for NAFLD are mostly based on weight reduction via energy restriction plus considering the manipulation of the proportion and type of macronutrients as well as dietary supplements.^13,14^

 Dietary Approaches to Stop Hypertension (DASH) diet – a plant-based dietary pattern and also life-long approach to healthy eating – has been designed for the prevention and treatment of hypertension.^15^ DASH diet includes low sodium, saturated fat and meat and emphasizes on consuming low-fat dairy products, vegetables and fruits, whole grains, fish, poultry and nuts, seeds and legumes that are high in protein, fiber, flavonoids, folate as well as potassium, calcium and magnesium.^15-17^ There is evidence indicating the protective effects of plant–based dietary pattern such as DASH or Mediterranean dietary pattern on improving metabolic biomarkers and anthropometric measures. For example, Arjmand et al^18^ found that 3-months following Mediterranean-DASH intervention for Neurodegenerative Delay (MIND) pattern on 37 obese women led to reduced anthropometric indices. It is supposed that DASH diet could be an effective approach in NAFLD prevention or treatment because adherence to DASH diet reduces liver fat accumulation as well as odds and risk of NAFLD.^19,20^ Moreover, there is limited interventional evidence indicating that adherence to DASH diet compared with usual weight loss diet revealed reductions in weight and serum liver enzymes, IR, lipid profile, inflammation and OS.^21^ DASH diet which is high in fruits and vegetables containing flavonoids with antioxidant activity could reduce free radicals and lipid peroxidation.^15^ Fruits and vegetables containing vitamins, minerals, and bioactive compounds such as antioxidants, various polyphenols and folate have been shown to be associated with inflammation and OS.^22^ Flavonoids (such as anthocyanidins, flavanols, flavanones, flavones, flavonols, and isoflavones) are the most common polyphenols responsible for antioxidant properties.^23^

 There is evidence illustrating that DASH diet seems to be more suitable dietary approach for metabolic conditions such as diabetes, cardiovascular disease and NAFLD compared with low-energy diets.^24^ Only one randomized clinical trial has been conducted to investigate the effects of 8 weeks DASH diet intervention on patients with NAFLD.^21^ Hence, due to the lack of interventional studies in examining the efficacy of DASH diet on NAFLD, this clinical trial aimed to assess the effect of DASH diet compared with low-calorie diet (LCD) on atherogenic indices, pro-oxidant-antioxidant balance as well as liver steatosis biomarkers and serum liver enzymes in patients with NAFLD.

## Materials and Methods

###  Setting of the study

 Sixty-two obese adults with NAFLD enrolled in the present double-blinded controlled randomized clinical. The participants were recruited through advertisements and referrals from physicians and families. The inclusion criteria were age between 20 and 50 years, 30 ≤ BMI < 40 kg/m^2^), newly diagnosed as grade I and II NAFLD. Those with grade III were excluded from the study. NAFLD was diagnosed based on ultrasonography findings (Sonoace X4 Medisio, South Korea). The severity of NAFLD was classified into grade I (mild) and II (moderate) according to Hamaguchi et al.^25^ Hepatic fatty infiltration grading was determined by considering the brightness of the liver, blurred vessels, contrast ratio of the liver-to-kidney and echogenicity.^25^

 Those who were alcohol drinkers, smoker, pregnant, breastfed, menopause, with regular exercise, following weight loss diet 3 months before the study, taking anti-diabetic, anti-lipidemic, anti-hypertensive, antibiotics, corticosteroids and oral contraceptives drugs, as well as with metabolic disease and chronic conditions such as type 2 diabetes, polycystic ovary syndrome, liver, kidney, thyroid, gastro-intestinal, autoimmune diseases and cancers were excluded.

###  Sample size

 The PAB concentration in patients with NAFLD reported by Nobakht Motlagh Ghoochani et al^26^ was applied for sample size calculation. The estimated sample size was 20 per each group based on a 95% confidence interval (CI) and %80 power using sample size software (PASS; NCSS, LLC, US). By considering a probable 10% dropout rate, the number reached to 22.

###  Randomization and blinding

 The subjects were randomly assigned in a 1:1 ratio to either the DASH or LCD group by an independent assistant not involved in the study using Random Allocation Software (RAS), in blocks stratified by gender (female vs male), age (18-35 yrs. vs 36-55 yrs.) and BMI status (< 35 kg/m^2^ vs ≥ 35 kg/m^2^)). All the participants and the assessors were blinded to the random allocation over the trial.

###  Dietary intervention

 Individual DASH and LCD diets were designed by a dietitian. Mifflin formula was applied to assess individually energy requirement and the prescribed DASH or LCD was considered as -500 Kcal from the estimated energy for all the patients.^27^ For both DASH and LCD diets, the proportion of carbohydrates, fat, and protein from energy were 55%-60%, < 30%, and 10%-15%, respectively. Based on the food-based dietary guidelines for Iranians (available at http://www.fao.org/nutrition/education/food-baseddietary-guidelines/regions/countries/iran/fr/), meal plan for LCD was prepared, whereas DASH diet was planned according to DASH dietary pattern.^28^

###  Assessment of dietary intake and physical activity 

 For dietary assessment, a 3-day food record (two non-consecutive weekdays and one weekend) was completed by the patients before and after the study. Daily energy and nutrient intakes were obtained using Nutrition IV software (First Databank: Hearst, San Bruno, CA, USA) at baseline and end of the study.

 Compliance to the LCD was checked based on the results of dietary assessment using a 3-day food records for each months as well as following the subjects through phone calls fortnightly.

 To confirm the adherence of DASH diet, Dixon’s DASH diet index was estimated according to Fung et al.^28,29^ All food components (groups) categorized into: (1) those food components which lower intakes are favorable (red and processed meats, sodium, sweetened beverages) and (2) the food groups which higher intakes are recommended (whole grains, vegetables, legumes, nuts and low-fat dairy products). The components were scored based on quintiles. The groups that lower intakes are favorable or high intakes are recommended were scored 1 and the rest were scored zero. The points were summed up to obtain the Dixon’s DASH diet index which ranged between 0 to 9.^30^

 Physical activity was checked based on estimating metabolic equivalent of task (MET-hours/week) score using the International Physical Activity Questionnaire-Short Form (IPAQ-SF) through face-to-face interview and categorized into: “low”, “moderate”, or “high” activity level at baseline and end of the trial.^31^

###  Anthropometric and blood pressure measurements

 Weight and height were measured wearing low clothes without shoes using stadiometer (Seca, Hamburg, Germany) with 100 g and 0.5 cm precision, respectively, and then, BMI was estimated. Waist circumference (WC) was also assessed at the halfway between the lower ribs and the iliac crest with precision of 0.1 cm. After 15 minutes resting, systolic blood pressure (SBP) and diastolic blood pressure (DBP) were measured in a seated position using an automated digital sphygmomanometer (Microlife A100–30, Berneck, Switzerland).

###  Laboratory assays

 Blood sample was taken from each patient after 12-14 hours overnight fasting and serum was separated after one hour. Lipid profile including triglyceride (TG), total cholesterol (TC), high and low- density lipoprotein cholesterol (HDL-C and LDL-C, respectively) as well as alanine aminotransferase (ALT) and aspartate aminotransferase (AST) concentrations were assessed at the same day immediately. At the end of the study, PAB was assessed according to Nobakht Motlagh Goochani et al^26^ using enzyme-linked immunosorbent assay (ELISA) technique and the values were expressed as arbitrary units (HK), i.e. percentage of hydrogen peroxide in the standard solution.

 AST to platelet ratio index(APRI) as an index for predicting liver fibrosis and lipid accumulation product (LAP) – as reliable biomarker for identifying individuals at immediate risk of cardiovascular diseases – were calculated.^32,33^

 LAP = [(WC-58)*TG(mmol/L] in men and [(WC-58)*TG(mmol/L) in women]

###  Study outcomes

 In the present study, changes in blood pressure and lipid profile, PAB, weight, WC and BMI were considered as primary outcomes while changes in atherogenic indices, liver enzymes and steatosis severity were defined as secondary outcomes.

###  Statistical analysis

 For the statistical analyses, IBM SPSS Statistics for Windows, version 26 (IBM Corp., Armonk, N.Y., USA) and per protocol principles were applied. After checking the normality of the distribution of continuous variables using Kolmogorov-Smirnov test, data were expressed by mean ± standard deviation (SD) or median (25^th^, 75th) for continuous variables and frequency (%) for categorical variables. Within-group changes were checked statistically using paired samples *t* test, Wilcoxon signed rank and also sign tests. Independent samples *t* test, Mann-Whitney U and chi-square tests were applied for between–group differences at baseline, respectively. At the end of the study, to control the confounders, the analysis of covariance (ANCOVA) and quantile regression were used to compare the changes between the groups. *P *values of less than 0.05 were considered statistically significant.

## Results

**Figure 1:**
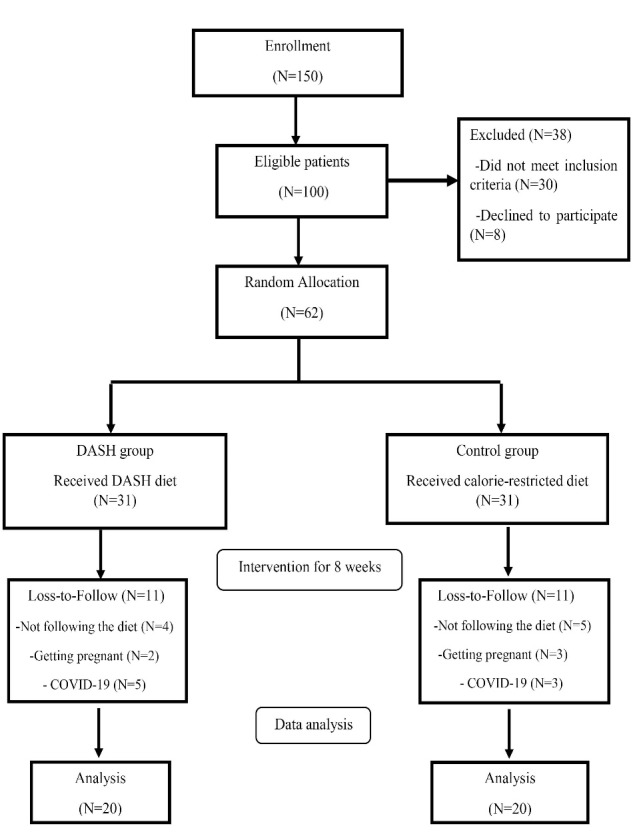
Study flow chart

Figure 1 shows the study flowchart based on the per-protocol analysis. Of 62 patients started the trial, 11 patients in each group lost to follow the trial because of un-related caused to the intervention and finally, 20 patients in each group completed the study.

Table 1 shows that no significant differences were observed in terms of personal details, weight and BMI, SBP, DBP, and the severity of liver steatosis between the groups at baseline.

**Table 1:** Baseline characteristic of the study participants

**Variable**	**DASH (n=20)**	**Control (n=20)**	***P***
Gender, No. (%)			0.594*
Female	13 (65.0)	12 (60.0)	
Male	7 (35.0)	8 (40.0)	
Marital status, No. (%)			0. 683*
Single	3 (15.0)	5 (25.0)	
Married	17 (85.0)	15 (75.0)	
NAFLD severity, No. (%)			0.751*
Mild	10 (50.0)	12 (60.0)	
Moderate	10 (50.0)	8 (40.0)	
Age (y), Mean ± SD	38.80 ± 9.98	37.10 ± 9.74	0.589**
Weight (kg), Mean ± SD	93.32 ± 19.51	93.49 ± 13.98	0.976**
Height (cm), Mean ± SD	166.49 ± 12.0	165.52 ± 7.86	0.764**
BMI (kg/m^2^), Mean ± SD	33.43 ± 4.09	34.02 ± 3.61	0.632**
SBP (mm Hg), Mean ± SD	130.68 ± 9.92	125.56 ± 12.47	0.156**
DBP (mm Hg), Mean ± SD	83.82 ± 10.41	82.78 ± 11.27	0.907**

DASH, Dietary Approaches to Stop Hypertension; NAFLD, Non-alcoholic fatty liver disease; BMI, Body mass index; SBP, Systolic blood pressure; DBP, Diastolic blood pressure. * *P* value for chi-square test. ** *P* value for independent sample *t* test.

 Daily dietary intake at baseline and after 8 weeks is presented inTable 2. No differences were observed in energy and macronutrient intakes.However,after 8 weeks,DASH groupshowed less saturated fat (*P* < 0.001) and selenium (*P* = 0.004) intakes and greater vitamins A (*P* = 0.014) and C (*P* = 0.023) intakes compared with control group.Moreover, Dixon’s DASH diet index increased from 8 to 9 in DASH group indicating good adherence to DASH diet.

**Table 2:** Daily dietary intakes before and after the study

**Variable**	**DASH (n=20)**	**Control (n=20)**	***P***
	**Mean±SD**	**Mean±SD**	
Energy (kcal)			
Baseline	1585.60 ± 187.67	1629.50 ± 206.54	0.488**
End	1553.00 ± 134.07	1640.10 ± 174.38	0.104***
*P* ^*^	0.326	0.895	
Carbohydrates (g)			
Baseline	227.55 ± 31.46	234.40 ± 36.20	0.527**
End	219.59 ± 22.94	232.80 ± 25.70	0.123***
*P* ^*^	0.243	0.949	
Protein (g)			
Baseline	73.09 ± 8.17	75.72 ± 12.51	0.436**
End	69.52 ± 8.94	70.85 ± 13.68	0.978***
*P* ^*^	0.192	0.164	
Fat (g)			
Baseline	46.17 ± 6.18	46.78 ± 10.11	0.841**
End	48.21 ± 5.61	49.84 ± 7.41	0.461***
*P* ^*^	0.094	0.270	
SFA (g)			
Baseline	9.37 ± 2.73	18.41 ± 5.66	< 0.001**
End	9.69 ± 2.00	17.39 ± 3.28	<0.001***
*P* ^*^	0.242	0.309	
MUFA(g)			
Baseline	21.09 ± 3.22	14.25 ± 3.99	<0.001**
End	21.63 ± 3.45	15.60 ± 3.48	0.057***
*P* ^*^	0.415	0.222	
PUFA (g)			
Baseline	8.89 ± 1.18	7.63 ± 4.45	0.227**
End	10.03 ± 1.93	9.82 ± 4.81	0.868***
*P* ^*^	0.088	0.096	
Cholesterol (mg)			
Baseline	158.71 ± 77.28	327.44 ± 100.40	0.022**
End	199.06 ± 107.71	367.52 ± 167.21	0.833***
*P* ^*^	0.291	0.158	
Dietary fiber (g)			
Baseline	23.06 ± 6.45	17.89 ± 5.57	0.010**
End	21.93 ± 6.32	17.19 ± 6.32	0.794***
*P* ^*^	0.065	0.346	
Phosphorus (mg)			
Baseline	1105.40 ± 259.63	973.53 ± 209.88	0.085^**^
End	1093.70 ± 260.32	898.74 ± 266.16	0.418^***^
*P* ^*^	0.214	0.346	
Calcium (mg)			
Baseline	856.98 ± 308.63	700.03 ± 269.69	0.095 ^**^
End	921.32 ± 291.93	592.40 ± 228.59	0.074^***^
*P* ^*^	0.357	0.062	
Magnesium (mg)			
Baseline	277.76 ± 65.74	227.29 ± 69.42	0.023^**^
End	266.94 ± 70.42	211.73 ± 70.25	0.705^***^
*P* ^*^	0.340	0.249	
Sodium (mg)			
Baseline	925.69 ± 344.87	1341.12 ± 504.75	**0.004** ^**^
End	987.39 ± 345.33	1277.44 ± 412.15	0.078^***^
*P* ^*^	0.465	0.536	
	**Median** **(25** ^th^ **, 75** ^th^ ** percentiles)**	**Median** **(25** ^th^ **, 75** ^th^ ** percentiles)**	
Vitamin A (RE)			
Baseline	1439.50 (520.63, 4114.50)	1690.00 (601.28, 3539.50)	1.000^b^
End	1776.00 (858.03, 5220.00)	702.95 (456.60, 1435.50)	**0.014** ^c^
*P* ^a^	**0.026**	0.199	
Vitamin E (mg)			
Baseline	4.11 (3.14, 5.05)	2.96 (2.32, 3.93)	0.063^b^
End	3.48 (2.34, 5.21)	3.08 (2.27, 3.81)	0.429^c^
*P* ^a^	0.570	0.472	
Vitamin C (mg)			
Baseline	176.75 (120.18, 248.23)	104.24 (67.30, 157.43)	**0.010** ^b^
End	151.60 (113.28, 236.90)	112.50 (79.69, 167.43)	**0.023** ^c^
*P* ^a^	**0.031**	0.913	
Zinc (mg)			
Baseline	8.67 (6.88, 10.30)	8.93 (7.86, 9.76)	1.000^b^
End	7.80 (6.53, 8.59)	8.28 (6.98,8.95)	0.547^c^
*P* ^a^	0.355	0.983	
Copper (mg)			
Baseline	1.43 (1.13, 1.66)	1.05 (0.92, 1.33)	**0.004** ^b^
End	1.24 (1.04, 1.34)	1.04 (0.92, 1.30)	0.056^c^
*P* ^a^	0.961	0.571	
Selenium (µg)			
Baseline	0.09 (0.06, 0.11)	0.09 (0.06, 0.13)	0.659^b^
End	0.07 (0.05, 0.09)	0.10 (0.07, 0.13)	**0.004** ^c^
*P* ^a^	0.404	0.199	

DASH, Dietary Approaches to Stop Hypertension; SFA, Saturated fatty acid; MUFA, Monounsaturated fatty acid; PUFA, Polyunsaturated fatty acid. Mean (Standard deviation) is presented for normally distributed data and median (25th and 75th percentiles), is presented for not normally distributed data. ^*^ *P* value for paired *t* test ^**^ *P* value for Independent samples *t* test. ^***^ *P* value for ANCOVA test (adjusted for baseline values). ^a^ *P* based on Wilcoxon signed-rank test. ^b^ *P* based on Mann–Whitney U test. ^c^ *P* based on Quantile regression adjusted for baseline values.

 Changes in anthropometric measures, atherogenic indices, serum liver enzymes and liver steatosis biomarkers as well as PAB in the study groups throughout the study are presented inTable 3.Comparing changes in anthropometric measures between the groups revealed that DASH group had greater reductions in weight (*P* = 0.021), BMI (*P* = 0.025) and WC (*P* = 0.002) than control group, after adjusting for weight change and baseline values. Although DBP and SBP significantly decreased in both group after 8 weeks, the reductions in DASH group were greater compared with control groups (-8.25 mm Hg vs -3.75 mm Hg and -12.25 mm Hg vs 5.25 mm Hg, respectively). However, results of inter-group difference in changes in DBP and SBP revealed no statistically significant differences, after adjusting for baseline values and weight change. Table 3 also illustrates statistically greater reductions in serum lipid profile (apart from HDL-C) as well as atherogenic indices (except for TG/HDL-C) in DASH group compared with control group, even after adjusting for baseline values and weight change.

**Table 3:** Anthropometric measures, atherogenic and liver steatosis biomarkers and PAB before and after the study

**Variable**	**DASH (n=20)**	**Control (n=20)**	***P***
	**Mean±SD**	**Mean±SD**	
Weight (kg)			
Baseline	93.32 ± 19.51	93.49 ± 13.98	0.976**
End	85.57 ± 18.62	87.88 ± 13.88	0.021***
MD (95% CI)	-7.75 (-9.34, -6.17)	-5.61 (-6.71, -4.50)	
*P* ^*^	**>0.001**	**<0.001**	
BMI (kg/m^2^)			
Baseline	33.43 ± 4.09	34.02 ± 3.61	0.632**
End	30.64 ± 4.06	31.96 ± 3.57	0.025***
MD (95% CI)	-2.79 (-3.35, -2.22)	-2.06 (-2.47, -1.64)	
*P* ^*^	**<0.001**	**<0.001**	
WC (cm)			
Baseline	111.25 ± 12.29	109.92 ± 9.80	0.708**
End	103.32 ± 12.67	105.0 ± 9.60	0.002***
MD (95% CI)	-7.92 (-9.58, -6.27)	-4.92 (-5.94, -3.91)	
*P* ^*^	**<0.001**	**<0.001**	
SBP (mm Hg)			
Baseline	131.25 ± 10.11	125.50 ± 11.91	0.156**
End	119.00 ± 9.54	120.25 ± 13.52	0.162***
MD (95% CI)	-12.25 (-17.38, -6.70)	-5.25 (-9.64, -0.86)	
*P* ^*^	**0.002**	**0.022**	
DBP (mm Hg)			
Baseline	83.50 ± 10.89	82.78 ± 11.27	0.907**
End	75.25 ± 5.73	77.35 ± 9.54	0.087***
MD (95% CI)	-8.25 (-13.01, -3.49)	-3.75 (-7.38, -0.19)	
*P* ^*^	**0.002**	**0.044**	
TG (mg/dL)			
Baseline	178.38 ± 73.04	136.23 ± 50.50	0.040**
End	117.05 ± 42.16	118.50 ± 49.50	0.037***
MD (95% CI)	-61.33 (-81.69, -40.96)	-17.73 (-31.22, -4.24)	
*P* ^*^	**<0.001**	**0.013**	
TC (mg/dL)			
Baseline	203.85 ± 49.84	173.35 ± 26.00	0.020**
End	164.20 ± 30.21	162.35 ± 24.63	0.011***
MD (95% CI)	-39.65 (-55.69, -23.61)	-11.00 (-18.48, -3.52)	
*P* ^*^	**<0.001**	**0.006**	
HDL-C (mg/dL)			
Baseline	48.55 ± 9.70	42.30 ± 8.61	0.037**
End	45.85 ± 9.54	41.90 ± 7.64	0.649***
MD (95% CI)	-2.70 (-6.25, 0.85)	-0.40 (-2.12, 1.33)	
*P* ^*^	0.128	0.636	
LDL-C (mg/dL)			
Baseline	124.77 ± 32.77	103.33 ± 27.16	0.030**
End	87.68 ± 20.54	92.22 ± 24.56	0.002***
MD (95% CI)	-32.09 (-47.29, -26.88)	-11.11 (-20.93, -1.27)	
*P* ^*^	**<0.001**	**0.029**	
Non-HDL-C (mg/dL)			
Baseline	155.30 ± 44.78	131.06 ± 25.56	0.044**
End	118.35 ± 26.65	120.45 ± 23.60	0.005***
MD (95% CI)	-36.95 (-52.52, -21.38)	-10.60 (-18.11, -3.10)	
*P* ^**^	**<0.001**	**0.008**	
TC/HDL-C			
Baseline	4.24 ± 0.83	4.24 ± 0.95	0.990**
End	3.67 ± 0.78	3.97 ± 0.78	0.033***
MD (95% CI)	-0.57 (-0.98, -0.16)	-0.27 (-0.51, -0.03)	
*P* ^*^	**0.009**	**0.028**	
TG/HDL-C			
Baseline	3.86 ± 1.79	3.42 ± 1.54	0.407**
End	2.69 ± 1.12	2.97 ± 1.46	0.129***
MD (95% CI)	-1.17 (-1.71, -0.64)	-0.45 (-0.83, -0.06)	
*P* ^*^	**<0.001**	**0.026**	
LDL-C/HDL-C			
Baseline	2.64 ± 0.75	2.55 ± 0.86	0.712**
End	1.97 ± 0.59	2.25 ± 0.64	0.009***
MD (95% CI)	-0.67 (-0.96, -0.38)	-0.30 (-0.56, -0.04)	
*P* ^*^	**<0.001**	**0.027**	
Non-HDL-C/HDL-C			
Baseline	3.24 ± 0.83	3.24 ± 0.95	0.990**
End	2.67 ± 0.78	2.97 ± 0.78	0.033***
MD (95% CI)	-0.57 (-0.98, -0.16)	-0.27 (-0.51, -0.03)	
*P* ^*^	**0.009**	**0.028**	
PAB (HK)			
Baseline	59.49 ± 21.84	55.05 ± 17.54	0.483**
End	60.08 ± 21.00	54.48 ± 16.94	0.293***
MD (95% CI)	0.59 (-12.44, 13.63)	-0.57 (-7.48, 6.33)	
*P* ^*^	0.925	0.864	
AST (IU/L)			
Baseline	24.10 ± 10.91	26.75 ± 9.28	0.413**
End	18.40 ± 6.57	25.05 ± 8.70	0.008***
MD (95% CI)	-5.70 (-9.42, -1.98)	-1.70 (-3.53, 0.13)	
*P* ^*^	**0.005**	0.067	
ALT (IU/L)			
Baseline	27.20 ± 14.0	37.35 ± 18.37	0.057**
End	18.75 ± 8.91	31.60 ± 16.24	0.149***
MD (95% CI)	-8.45 (-12.89, -4.01)	-5.75 (-10.46, -1.04)	
*P* ^*^	**0.001**	**0.019**	
AST/ALT			
Baseline	0.97 ± 0.38	0.81 ± 0.28	0.163**
End	1.08 ± 0.34	0.89 ± 0.30	0.651***
MD (95% CI)	0.12 (-0.09, 0.32)	0.08 (0.01, 0.15)	
*P* ^*^	0.242	**0.031**	
Ferritin (ng/ml)			
Baseline	109.55 ± 124.34	94.42 ± 82.74	0.174**
End	91.76 ± 98.27	84.83 ± 2.36	
MD (95% CI)	-17.79 (-40.87, -5.29)	-9.60 (-30.37, 11.18)	0.688***
*P* ^*^	0.123	0.346	
Liver fibrosis score			
Baseline	-2.05 ± 1.18	-2.72 ± 1.44	0.114**
End	-2.27 ± 1.00	-2.82 ± 1.41	0.156***
MD (95% CI)	-0.22 (-0.48, 0.04)	-0.10 (-0.29, 0.08)	
*P* ^*^	0.126	0.356	
APRI			
Baseline	0.32 ± 0.19	0.34 ± 0.17	0.708**
End	0.25 ± 0.11	0.33 ± 0.16	0.019***
MD (95% CI)	-0.07 (-0.12, -0.01)	-0.01 (-0.03, 0.01)	
*P* ^*^	**0.016**	0.225	
LAP			
Baseline	455.12 ± 206.96	339.46 ± 144.36	0.047**
End	248.38 ± 107.10	266.44 ± 137.86	0.003***
MD (95% CI)	-206.74 (-266.96, -146.51)	-73.01(-101.50, -44.54)	
*P* ^*^	**<0.001**	**<0.001**	

DASH, Dietary Approaches to Stop Hypertension; BMI, Body mass index; WC, Waist circumference; DBP, Diastolic blood pressure; SBP, Systolic blood pressure; TG, Triglyceride; TC, Total cholesterol; HDL-C, High-density lipoprotein cholesterol; LDL-C, Low-density lipoprotein cholesterol; PAB, prooxidant- antioxidant balance; HK, Hamidi-Koliakos Arbitrary Unit Based on the Percentage of Hydrogen Peroxide Evaluated in Standard Solution; AST, Aspartate aminotransferase; ALT, Alanine aminotransferase; APRI, Aspartate aminotransferase to platelet ratio index; LAP, Lipid accumulation product; MD, mean difference. * *P* value for paired *t* test. ** *P* value for independent samples *t *test. ***P value for ANCOVA test (adjusted for baseline values).

 Serum levels of AST, ALT, AST/ALT ration as well as liver fibrosis score, APRI and LAP decreased greater in DASH group than control group, nevertheless, after adjusting for baseline values and weight change, only the reductions in serum AST, APRI and LAP did reach to statistically significant level (*P* = 0.008, *P* = 0.019 and *P* = 0.003, respectively). Moreover, changes in serum PAB level revealed no statistically significant difference between the groups at the end of the study (*P* = 0.293).

**Figure 2:**
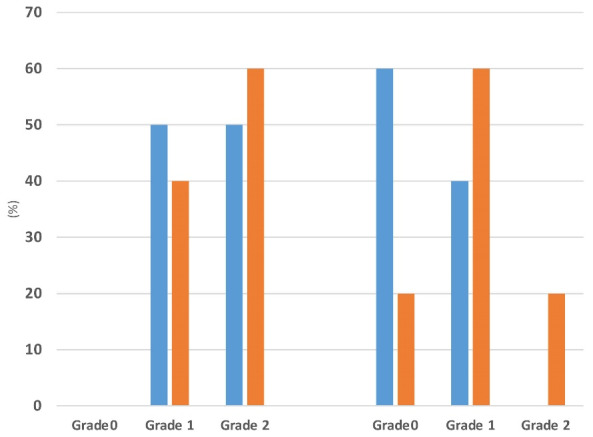
The effectiveness of adherence to DASH diet on liver steatosis. Intra-group comparison using sign test (*P* < 0.001 in DASH group and *P* 0.005 in control group, respectively). Inter-group comparison using chi-square test (*P* = 0.751 at baseline and *P* = 0.009 at the end of the study)

 The effectiveness of adherence to DASH diet on liver steatosis is presented in Figure 2. Although no significant differences were observed in liver steatosis severity between the groups at the beginning of the trial, 80% and 15% of patients in DASH group showed one grade (relative improvement) and two grade (complete improvement) reductions in liver steatosis whereas of those in control group, 20% demonstrated one grade reduction and 60% showed no change in liver steatosis (*P* = 0.012).

## Discussion

**Figure 3:**
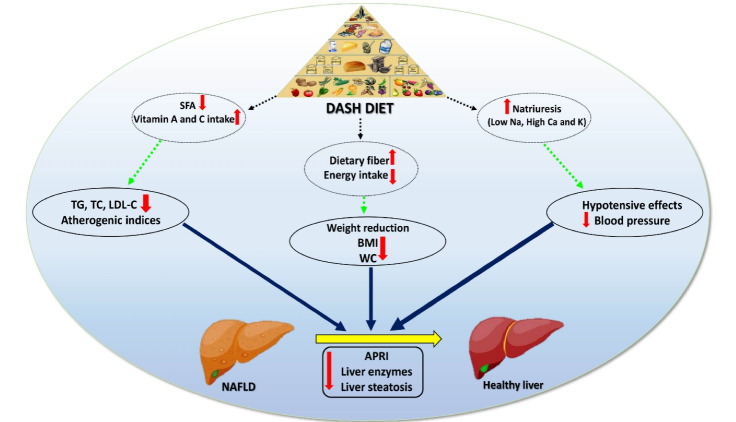
The effects of DASH diet on obesity, lipid profile, atherogenic indices and liver function in NAFLD. Adherence to DASH diet seems to be efficient in improving obesity by reducing body weight, BMI and WC, improving lipid profile (TG, TC, LDL-C) and atherogenic indices. Taken together the synergic aforementioned improvements, emerge as alleviated liver enzymes and reduced steatosis in patients with NAFLD. Abbreviations: APRI, AST to platelet ratio index; BMI, Body mass index; Ca, Calcium; DASH, Dietary Approaches to Stop Hypertension; K, Potassium; LDL-C, Low-density lipoprotein cholesterol; Na, Sodium; NAFLD, non-alcoholic fatty liver disease; SFA, Saturated fatty acids; TC, Total cholesterol; TG, Triglycerides; WC, Waist circumference

 Recently, a great attention has been paid for DASH dietary pattern as a dietary approach in prevention and/or treatment of NAFLD through cross-sectional, case-control and interventional studies. Previous studies have reported an inverse association between adherence to DASH- dietary pattern and NAFLD.^19-21^ Our results showed that adherence to DASH diet in comparison to usual LCD was more effective in improving anthropometric measures, atherogenic and liver steatosis biomarkers but not OS. Possible mechanistic effects of DASH diet in the treatment of NAFLD are shown in Figure 3.

 Daily dietary intake at baseline and after 8 weeks (Table 2) were not different in terms of energy and macronutrient intakes, however,patients in DASH groupconsumed less saturated fat and selenium intakes and greater vitamins A and C compared with control group.Moreover, studied patients in DASH group had good adherence to DASH diet based on Dixon’s DASH diet index at the end of the study which was 9 (ranged 0-9). Therefore, changes in the study outcomes could be attributed to the weight loss intervention diet.

 Our results revealed greater reductions in weight, WC and BMI in DASH group compared with control group (Table 3). There is cumulative evidence in weight- and WC-lowering effects of DASH dietary pattern.^24,34,35^ In a systematic review and meta-analysis by Soltani et al ^24^ results showed that adults on DASH diet lose more weight (weighted mean difference 1.42 kg (95% CI: 2.03, 0.82) in 8–24 weeks, BMI 0.42 kg/m^2^ (95% CI: 0.64,0.20) in 8–52 weeks and WC = 1.05 cm (95% CI: 1.61, 0.49) in 24 weeks compared with controls. Perry et al^34^ reported that the greatest contributing factor to the reduction in total body weight was the loss of body fat. The underlying mechanism is related to the DASH diet contents. DASH dietary pattern emphasizes on increased intakes of fruit, vegetables, fat-free/low-fat dairy, whole grains, nuts and legumes, and in contrast, limited intakes of saturated fat, cholesterol, red and processed meats, sweets, salt, sweets, added sugars and sugar-sweetened beverages.^15^ One of the main contributing factor in weight reduction could be greater intake of dietary fiber.^36^ Foods with high fiber influence chewing time, gastric distension, sense of feeling, time for digestion and absorption of nutrients and therefore, could increase satiety and delay hunger and resulted in lower energy intake and weight reduction.^36,37^

 Both DBP and SBP in this study significantly decreased in both groups (Table 3). Although there were greater reductions in DASH group (-8.25 mm Hg and -12.2 mm Hg, respectively) than control group (-3.75 mm Hg and -5.25 mm Hg, respectively) but inter-group difference was not statistically significant, after adjusting for baseline values and weight change. There is a wider body of evidence showing BP-lowering effect of DASH dietary pattern through natriuretic action as well as interacting with the renin–angiotensin–aldosterone system which lead to vascular and hormonal responses, and in turn, causes a hypotensive effect.^30,38^ These effects assumed to be related with low sodium intake and also high potassium or calcium.^16,24^

 Regarding lipid profile and atherogenic biomarkers, we found, apart from HDL-C, DASH diet resulted in marked decreases in serum lipid profile and atherogenic indices (except for TG/HDL-C) compared with control group, even after adjusting for baseline values and weight change. An umbrella review of systematic reviews and meta-analysis on the effect of DASH diet on cardiometabolic factors has reported cholesterol-lowering effect (particularly TC and LDL-C) of adherence to DASH diet and suggested it for reduction in cardiovascular diseases.^39^ Increasing evidence recommends plant-based dietary patterns such as DASH diet as these dietary pattern emphasize on fruits, vegetables, whole grains, legumes, seeds with less red or processed meats, high-fat foods, sugar-content foods and beverages.^15^ DASH diet also includes high-fiber foods with low glycemic index which are not energy-dense with cholesterol-lowering effects through delaying gastric emptying and slowing absorption of macronutrients.^38,39^

 Changes in serum PAB level-as an index for balance of pro-oxidants and antioxidants- revealed no statistically significant difference between the groups after controlling for the confounders at the end of the study (*P* = 0.293). The enzymatic antioxidants such as [catalase, glutathione peroxidase, superoxide dismutase] and non-enzymatic antioxidants (such as vitamin A, C, E, polyphenols and flavonoids) are mostly investigated separately.^26^ PAB which assays oxidants (such as malondialdehyde, catalase, glutathione peroxidase, superoxide dismutase) and antioxidants simultaneously in one single test is a relatively simple, rapid, and cheap technique.^40^ There is cumulative evidence either assessing OS using PAB in different conditions or the effect of DASH diet on OS.^41-44^ However, studies investigating PAB level in patients with NAFLD are very limited and are cross-sectional or case-control studies demonstrating that either patients with NAFLD had higher PAB level.^26,45^ To the best of our knowledge, there is only one study investigated the effect of DASH diet on NAFLD. Mirhafez et al^46^ studied the effect of curcumin with piperine supplementation on PAB in patients with NAFLD showed that curcumin but not co-supplemented curcumin had significantly PAB-lowering effect. Another study by reviewing the literature illustrated a good evidence of the BP-lowering effect of bioactive compounds such as lycopene, docosahexaenoic acid, and dietary fiber which are high in DASH dietary pattern.^47^ Indeed, a systematic review and meta-analysis in 2020 indicates that DASH diet could significantly increase glutathione and decrease malondialdehyde levels, with a trend to improve total antioxidant capacity (TAC), nitric oxide, and f2-isoprostanes by the adherence to the DASH diet.^48^

 NAFLD is a well-known leading cause of liver transplantation in men and the second in women. Elevated liver enzymes and hepatic fat accumulation are characteristics of NAFLD.^38^ Greater reductions in serum AST, APRI and LAP were found in DASH group than control group. Moreover, 80% and 15% of patients in DASH group showed one grade (relative improvement) and two grade (complete improvement) reductions in liver steatosis. Similar results regarding beneficial effects of DASH diet on serum liver enzymes and also liver steatosis and fibrosis were reported.^49-52^ It appears that DASH diet because of high dietary fiber content and its effect on satiety, weight and WC as well as glucose and lipid-lowering effect could improve liver steatosis and fibrosis. However, to best of knowledge, there are limited interventional studies investigating the effect of DASH diet on liver function biomarkers, particularly liver fibrosis score, APRI as well as LAP for prediction of the cardiovascular risk in NAFLD.

 The present study had some limitations such as short follow-up, lack of assessing other oxidative and anti-oxidative biomarkers as well as more reliable indicators of liver fibrosis and steatosis. However, considering PAB as a simple and easy test to measure both oxidative and anti-oxidative status, prescribing LCD as an approved strategy for NAFLD obese patients without receiving any medication or treatment, providing DASH diet based on the individual habitual diet and estimating APRI and LAP indices are considered as the study strengths.

## Conclusion

 According to the results of the present study, adherence to DASH diet appears to be more effective in improving obesity, atherogenic and liver steatosis biomarkers but not OS than usual LCD. However, further clinical trials are encouraged to study the effect of DASH diet in patients with NAFLD in long-term.

## Acknowledgements

 We sincerely thank the patients who participated in the present study.

## Competing Interests

 The authors declare no conflict of interest in publishing this paper. This study not supported by any grant money from a pharmaceutical company or for-profit organization.

## Ethical Approval

 This trial was performed in accordance with the guidelines of the Declaration of Helsinki and approved by the Ethics Committee of Research vice-chancellor (Ethics code: TBZMED. REC.1400.009) as well as registered in the Iranian Registry of Clinical Trials (IRCT20100209003320N17, https://www.irct.ir/trial/42625**)**. All patients completed and signed a consent form after describing study objectives and protocol.

## Funding

 This study was funded by the ‘Research Vice-Chancellor’ of Tabriz University of Medical Sciences, Tabriz, Iran. This paper is a part of the data obtained from an MSc dissertation submitted to Tabriz University of Medical Sciences (Taghi Badali).
